# Ectoine Inhibits IL-1β-Induced Inflammation by Suppressing the NF-κB Pathway in Chondrocytes and Alleviates Osteoarthritis in a Rat Model

**DOI:** 10.3390/biomedicines14081756

**Published:** 2026-08-04

**Authors:** Peng Li, Ping Xie, Lishuai Miao, Mingdong Li, Zhiqi Zhu

**Affiliations:** 1 Orthopedic Surgery Department of The Second Affiliated Hospital, School of Medicine, The Chinese University of Hong Kong, Shenzhen & Longgang District People’s Hospital of Shenzhen, Shenzhen 518172, China; mlshaoyisheng@163.com; 2 Pathology Department of The Second Affiliated Hospital, School of Medicine, The Chinese University of Hong Kong, Shenzhen & Longgang District People’s Hospital of Shenzhen, Shenzhen 518172, China; xpp0223@163.com; 3 Department of Orthopedics and Traumatology, Hainan General Hospital, Hainan Affiliated Hospital of Hainan Medical University, Haikou 570311, China

**Keywords:** osteoarthritis, rats, chondrocytes, NF-κB, IL-1β

## Abstract

**Background:** Osteoarthritis (OA) is a degenerative joint disease characterized by inflammation and cartilage destruction, partly mediated by interleukin (IL)-1β-induced nucle factor (NF)-κB activation. Ectoine (Ec) is a natural osmoprotectant with anti-inflammatory properties; however, its effects on NF-κB signaling in OA remain unclear. This study investigated whether ectoine attenuates IL-1β-induced inflammation in chondrocytes by suppressing NF-κB activation and mitigates OA progression in a rat model. **Methods:** Primary rat chondrocytes were pretreated with ectoine (0–3.0% *w*/*v*) and then stimulated with IL-1β (10 ng/mL). Cell viability was evaluated. RT-qPCR and Western blotting were used to determine the expression of inflammatory markers (inducible nitric oxide synthase [*iNOS*], cyclooxygenase [*COX*]-2, tumor necrosis factor [*TNF*]-α, and matrix metalloproteinase [*MMP*]-3/13), and NF-κB pathway activity was assessed through p65 phosphorylation and inhibitor of NF-κB alpha (IκBα) degradation. In vivo, OA was induced using the modified Hulth method, followed by intra-articular injection of ectoine alone or combined with hyaluronic acid (HA). Cartilage integrity was assessed using Osteoarthritis Research Society International (OARSI) scoring at 8 weeks. **Results:** Ectoine at 1.5% significantly inhibited IL-1β-induced NF-κB activation, reducing p65 phosphorylation by 59% and IκBα degradation by 41%. This inhibition decreased proinflammatory mediators (iNOS 43%, COX-2 35%, TNF-α 41%) and matrix-degrading enzymes (MMP-3 23%, MMP-13 31%), while increasing type II collagen by 84%. In vivo, ectoine reduced cartilage erosion (OARSI score: 7.0 vs. 10.2 in OA group). The Ec–HA combination improved cartilage retention by 43% compared with ectoine alone. **Conclusions:** These preclinical findings suggest that ectoine was associated with reduced NF-κB activation markers and attenuated OA-like changes in rat models. The enhanced effect observed with HA supports further investigation of combined therapeutic strategies for OA management.

## 1. Introduction

Osteoarthritis (OA) is a chronic joint disorder marked by articular cartilage degradation and secondary bone changes, resulting in pain and functional impairment [1]. Importantly, OA is now recognized as a whole-joint disease involving pathological changes in all joint tissues, including meniscal degeneration and inflammation of both the infrapatellar fat pad and the synovial membrane [2]. Although OA has a multifactorial etiology, inflammation, especially that mediated by cytokines such as interleukin (IL)-1β, is strongly involved in its progression. Reportedly, IL-1β activates signaling pathways, including nuclear factor (NF)-κB, which upregulate inflammatory and catabolic genes that promote cartilage breakdown [3,4].

Ectoine (Ec), 1,4,5,6-tetrahydro-2-methyl-4-pyrimidine carboxylic acid (C_6_H_10_N_2_O_2_), is a natural compound produced by halophilic bacteria to protect cells against osmotic stress [5]. In addition to its stabilizing effects, ectoine has shown anti-inflammatory activity in models of mucosal and epithelial inflammation [6,7,8]. Protective effects of ectoine on chondrocytes have been reported previously [9], but the underlying mechanisms, particularly those involving NF-κB signaling in OA, remain elusive.

Present OA treatments, including nonsteroidal anti-inflammatory drugs (NSAIDs), intra-articular corticosteroids, and hyaluronic acid (HA), mainly relieve symptoms but do not stop disease progression and may cause notable side effects (e.g., gastrointestinal, cardiovascular, or renal risks with long-term NSAID use; possible cartilage damage after repeated corticosteroid administration; and local injection-site reactions, with limited efficacy in advanced disease for HA) [10,11]. This absence of disease-modifying therapies emphasizes the need for safer and more targeted strategies.

Given the anti-inflammatory profile and safety of ectoine, this study aimed to investigate its ability to inhibit IL-1β-induced inflammation and cartilage degradation in chondrocytes through modulation of the NF-κB pathway in a rat OA model.

## 2. Materials and Methods

### 2.1. Animals

In total, 64 Sprague-Dawley rats (32 males, 32 females; aged 8 weeks [*n* = 60] and 4 weeks [*n* = 4]) were obtained from Zhuhai Bestest Biotechnology Co. (production license: SYXK(Y) 2020-0230) for cell culture and OA modeling. Sample size was determined a priori by formal power analysis using G*Power 3.1 software (α = 0.05, 1-β = 0.80, and effect size = 0.5; Düsseldorf, Germany). Animals were randomly allocated using a computer-generated randomisation sequence. Both male and female rats were evenly distributed among all treatment groups. The rats were housed at the Experimental Animal Center of Longgang District People’s Hospital of Shenzhen under controlled conditions (23 ± 2 °C, 40–60% relative humidity) with *ad libitum* access to food and water. The animal experimental protocol was approved by the Ethics Committee of Longgang District People’s Hospital of Shenzhen (ethics record number: 2021005DW, 8 March 2021) and was conducted in accordance with the ARRIVE guidelines and AVMA Guidelines for the Euthanasia of Animals.

### 2.2. Isolation and Culture of Chondrocytes

Herein, 4-week-old Sprague–Dawley rats (2 males, 2 females) were euthanized by inhalation of an overdose of 5% isoflurane. Knee joint cartilage was isolated under aseptic conditions, minced, and sequentially digested with 0.25% trypsin (C0201; Beyotime, Shanghai, China) for 30 min and 0.2% type II collagenase (C222; Sigma, St. Louis, MO, USA) for 1 h. The digested tissue was filtered through a 200-mesh screen, centrifuged at 500× *g* for 8 min, and resuspended in Dulbecco’s modified Eagle medium supplemented with 10% fetal bovine serum (16140089; Gibco, Grand Island, NY, USA) and 1% penicillin/streptomycin (100×, C022; Beyotime, Shanghai, China). Cells were cultured at 37 °C in a 5% CO_2_ incubator. When 80% confluence was reached, chondrocytes were passaged at a 1:3 ratio using 0.25% trypsin. Second-passage cells were used in subsequent experiments.

### 2.3. Preparation of Ectoine Solutions

Ectoine (50, 100, 150, and 300 mg; purity > 99%; SIYOMICRO, Shenzhen, China) was dissolved in culture medium (10 mL) to prepare 0.5%, 1.0%, 1.5%, and 3.0% (*w*/*v*) solutions, followed by filtration through 0.22-μm sterile filters (Millipore, Burlington, MA, USA).

### 2.4. Cell Viability Assay

Chondrocyte viability was evaluated using the 3-(4,5-dimethylthiazol-2-yl)-2,5-diphenyltetrazolium bromide (MTT) assay. Cells were seeded in 96-well plates at 5000 cells/well and treated with 0.5%, 1.0%, 1.5%, or 3.0% (*w*/*v*) ectoine for 24 and 48 h. After incubation with MTT (5 mg/mL; Beyotime, Shanghai, China) for 4 h, formazan crystals were dissolved in dimethyl sulfoxide (D8418; Sigma, St. Louis, MO, USA), and absorbance was measured at 490 nm using a microplate reader (SYNERH1, BioTek, Winooski, VT, USA). Cell viability was calculated as follows:

Viability (%) = ([OD experiment − OD zero]/[OD control − OD zero]) × 100

### 2.5. In Vitro and In Vivo OA Models

For in vitro studies, chondrocytes seeded in six-well plates (5 × 10^4^ cells/mL) were treated with 0.5%, 1.0%, or 1.5% (*w*/*v*) ectoine solution, followed by IL-1β treatment (10 ng/mL; I2393; Sigma, St. Louis, USA) at 37 °C for 24 h after 80% confluence was reached, except in the blank control. For in vivo studies, OA was induced in sixty 10-week-old Sprague–Dawley rats (30 males and 30 females) using the modified Hulth method (for resection of the medial collateral ligament and excision of the medial meniscus and anterior cruciate ligament), as described previously [12]. All surgical procedures were performed by a single experienced surgeon. Inclusion criteria required successful surgery without fracture, joint dislocation, or infection; animals with excessive bleeding or failed meniscal transection were to be excluded (in practice, none met these criteria). All rats received buprenorphine subcutaneously (0.05 mg/kg; Tianjin Institute of Pharmaceutical Research, Tianjin, China) immediately after surgery and every 12 h during the first 48 h after surgery. The rats were randomly assigned to the following five groups (*n* = 12/group): sham, modeling (OA), ectoine, HA (600,000–1,500,000 Da; 10 mg/mL; Bausch & Lomb Freda, Jinan, China), and Ec + HA. From the third week after surgery, 50 μL of 1.5% ectoine, HA, or Ec + HA was administered weekly by intra-articular injection for 4 weeks. The sham and OA groups received the same dose of physiological saline. During the postoperative period, animals were monitored daily for signs of pain, distress, or infection; no adverse events or unexpected deaths were observed, and no animals were excluded after surgery. All 60 rats were euthanized at 8 weeks after surgery through inhalation of 5% isoflurane for >3 min using a gradual fill method to minimize distress, and their knee joint specimens (*n* = 60) were collected for histological and molecular analyses. Death was confirmed by absence of heartbeat (via cardiac palpation for at least 8 min) and cervical dislocation as a secondary physical method.

### 2.6. Reverse Transcription–Quantitative Polymerase Chain Reaction (RT-qPCR)

RT-qPCR was used to quantify mRNA expression of inducible nitric oxide synthase (*iNOS*), cyclooxygenase (*COX*)-2, matrix metalloproteinase (*MMP*)-3, *MMP*-13, tumor necrosis factor (*TNF*)-α, *IL-1β*, *aggrecan*, and collagen type II alpha-1 (*Col2A1*) in chondrocytes and femoral condyle samples. Total RNA was extracted using TRIzol reagent (R401; Vazyme, Nanjing, China), and complementary DNA was synthesized using the PrimeScript™ RT reagent Kit (RR037A; Takara, Japan). Reactions were performed using Taq Pro Universal SYBR qPCR Master Mix (Q712; Vazyme, Nanjing, China) and a QuantStudio3 Real-Time PCR System (Applied Biosystems, Foster City, CA, USA), with the following cycling conditions: predenaturation at 95 °C for 30 s, followed by 40 cycles of 95 °C for 5 s and 60 °C for 30 s. Gene expression was normalized to β-actin and analyzed using the 2^−ΔΔCq^ method. Primers (SIYOMICRO, Shenzhen, China) used in this study are listed in Table 1.

### 2.7. Western Blotting

Protein levels of iNOS, COX-2, MMP-1, MMP-3, MMP-13, p65, phosphorylated p65, inhibitor of NF-κB alpha (IκBα), and phosphorylated IκBα were determined by Western blotting. Total protein was extracted, quantified using a bicinchoninic acid protein assay kit, and electrophoresed on precast sodium dodecyl sulfate-polyacrylamide gels (P0052A/P0052B; Beyotime, Shanghai, China). Membranes were blocked with 5% nonfat dry milk for 2 h at room temperature, and then incubated overnight at 4 °C with primary antibodies (Servicebio, Wuhan, China; 1:1000 dilution, except β-actin at 1:5000). Next, membranes were incubated with horseradish peroxidase-conjugated goat anti-rabbit secondary antibody (1:3000) for 2 h at room temperature. Bands were detected using an enhanced chemiluminescence kit and quantified using ImageJ 1.5 software (NIH, Bethesda, MD, USA).

### 2.8. Histological Assessment

Rats were euthanized at 8 weeks after surgery, and knee joints were dissected into tibial plateau and femoral condyle sections. Following the OARSI histopathology guidelines for rats, medial tibial plateau tissues underwent safranin O-fast green staining and immunohistochemistry (IHC) to evaluate the therapeutic potential of ectoine against cartilage degeneration in the rat OA model. From each animal, three non-adjacent sections were selected from the most severely affected regions. Cartilage damage was quantified using the Osteoarthritis Research Society International (OARSI) scoring system, and the mean value of the three selected sections was used [13]. Femoral condyle articular cartilages were analyzed by RT-qPCR to assess the genetic regulatory effects of ectoine on cartilage degradation.

For IHC, paraffin-embedded sections were dewaxed, rehydrated, and subjected to antigen retrieval with pepsin (20 min). Endogenous peroxidase was quenched with 3% H_2_O_2_, and sections were blocked with 3% bovine serum albumin (30 min, room temperature). The sections were then incubated overnight at 4 °C with primary antibodies (Servicebio, Wuhan, China) against type II collagen (1:200) and p65 (1:200). After incubation with secondary antibody (1:200, 50 min, room temperature), immunoreactivity was visualized using 3,3′-diaminobenzidine and counterstained with Mayer’s hematoxylin. Quantification was performed through microscopy and ImageJ software.

### 2.9. Statistical Analyses

Experiments were performed at least three times using samples from different rats, and data are expressed as mean ± standard deviation. For histological and biochemical outcomes, all assessments were performed by two independent observers blinded to group allocation, and blinding was maintained until final data analysis. For OARSI scoring, disagreements between observers were resolved by consensus. Normality of the data was assessed using the Shapiro–Wilk normality test and the results showed no significant deviation from normality. Thus, one-way analysis of variance was applied to RT-qPCR, Western blotting, cell viability, IHC, and OARSI score data, with Tukey post hoc tests for pairwise comparisons. All statistical analyses were performed using GraphPad Prism 9 (GraphPad Software, San Diego, CA, USA). Statistical significance was set at *p* < 0.05.

## 3. Results

### 3.1. Ectoine Enhanced Chondrocyte Viability

**Figure 1:**
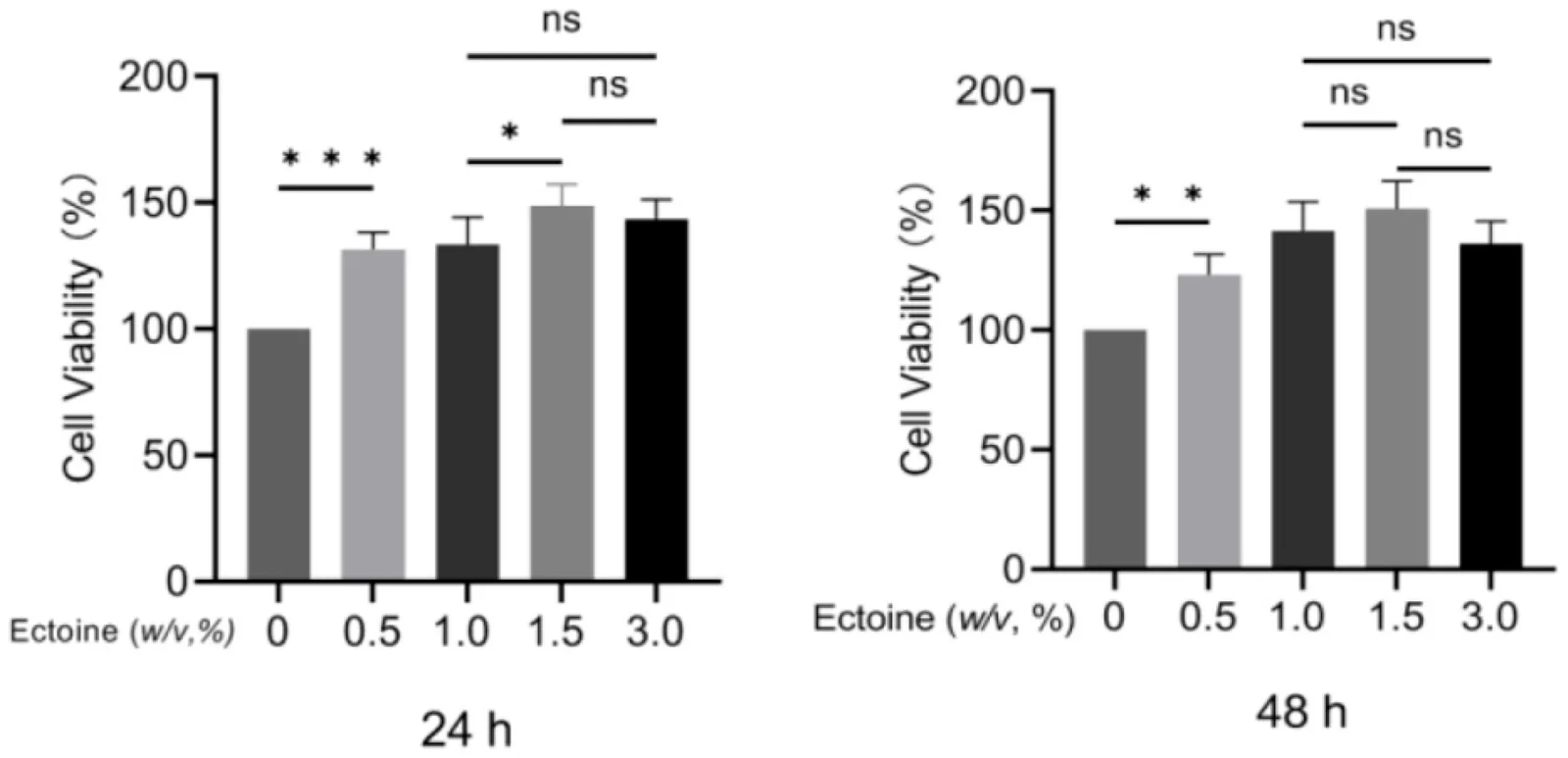
Ectoine enhanced chondrocyte viability. Ectoine enhanced chondrocyte viability in a dose-dependent manner within 0–1.5%, whereas a higher concentration (3%) reduced this effect (*n* ≥ 3, one-way *ANOVA*, ns, *p* > 0.05; * *p* < 0.05, ** *p* < 0.01, *** *p* < 0.001).

Ectoine enhanced chondrocyte viability in a dose-dependent manner within 0–1.5%, whereas a higher concentration (3%) reduced this effect (Figure 1). Compared with the control group, ectoine concentrations of 0.5–3.0% significantly increased viability at 24 and 48 h (*p* < 0.01). However, viability in the 3% group was not significantly different from that in the 1.0% or 1.5% groups and was lower than that in the 1.5% group, suggesting concentration-dependent saturation or cytotoxicity. Based on these results, ectoine concentrations of 0.5%, 1.0%, and 1.5% were used in subsequent experiments.

### 3.2. Ectoine Inhibited IL-1β-Induced Inflammatory Marker Expression and Preserved Type II Collagen

**Figure 2:**
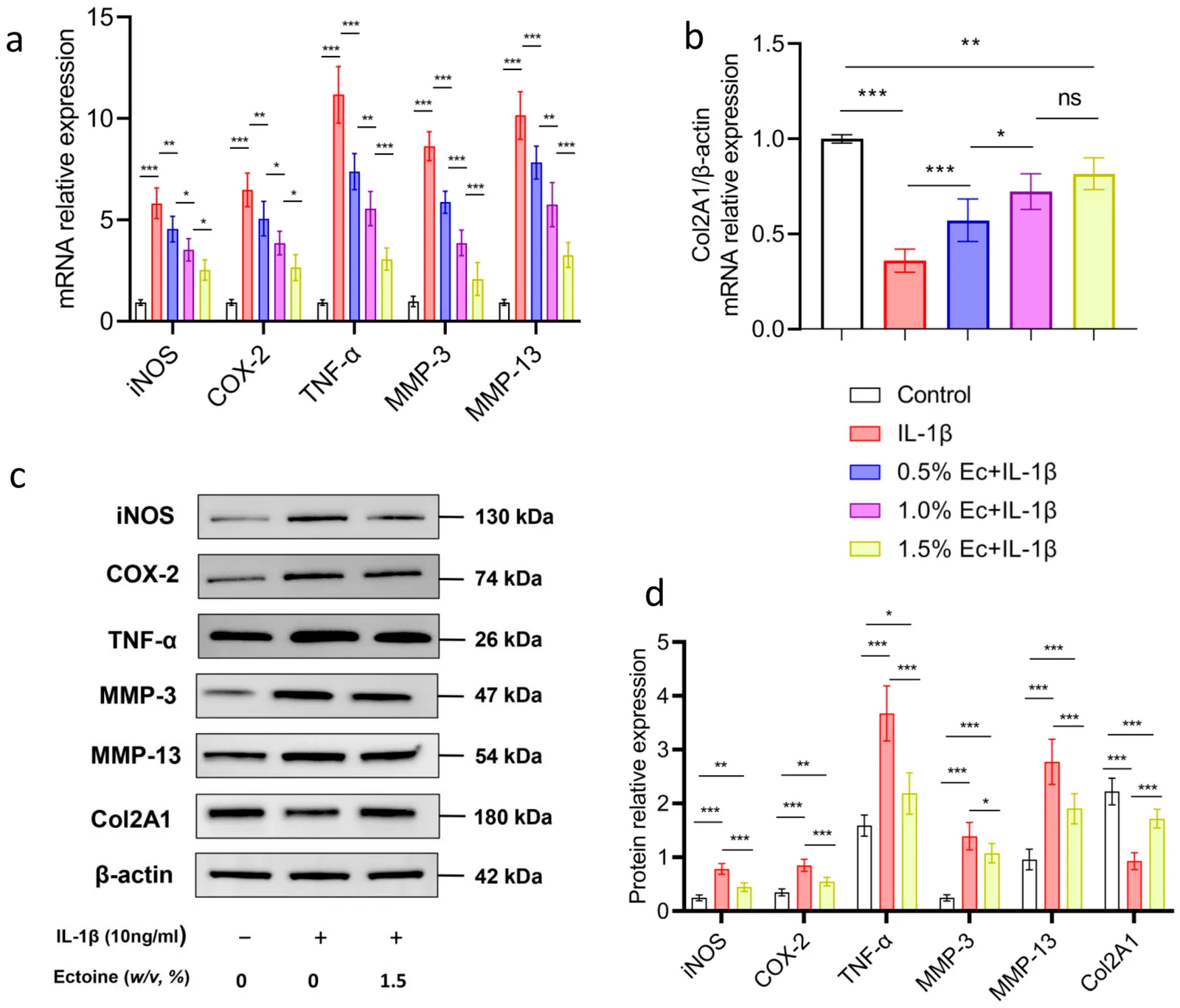
Ectoine inhibited IL-1β-induced inflammatory marker expression and preserved type II collagen. (**a**,**b**) Relative expression of *iNOS*, *COX-2*, *TNF-α*, *MMP-3*, *MMP-13* and *Col2A1* genes. (**c**,**d**) Western blots of these proteins in different groups (the samples derive from the same experiment and that blots were processed in parallel) (*n* ≥ 3, one-way *ANOVA*, ns, *p* > 0.05; * *p* < 0.05, ** *p* < 0.01, *** *p* < 0.001).

Compared with the control group, IL-1β stimulation markedly upregulated inflammatory mediators (*iNOS*, *COX-2*, *TNF-α*, *MMP-3*, *MMP-13*) in chondrocytes (*p* < 0.001), confirming successful OA model induction. Ectoine pretreatment reduced these markers in a dose-dependent manner (*p* < 0.001), with 1.5% ectoine showing the strongest effect (Figure 2a). RT-qPCR further showed higher *Col2A1* expression in ectoine-treated groups than in the IL-1βgroup (*F* = 49.40, *p* < 0.001; Figure 2b).

Western blotting supported these gene expression findings. Pretreatment with 1.5% ectoine reduced IL-1β-induced protein levels of proinflammatory mediators (iNOS by 43%, COX-2 by 35%, TNF-α by 41%) and cartilage-degrading enzymes (MMP-3 by 23%, MMP-13 by 31%), while increasing type II collagen by 84% relative to the IL-1β group (*p* < 0.05; Figure 2c,d).

### 3.3. Ectoine Suppressed IL-1β-Mediated NF-κB Pathway Activation

**Figure 3:**
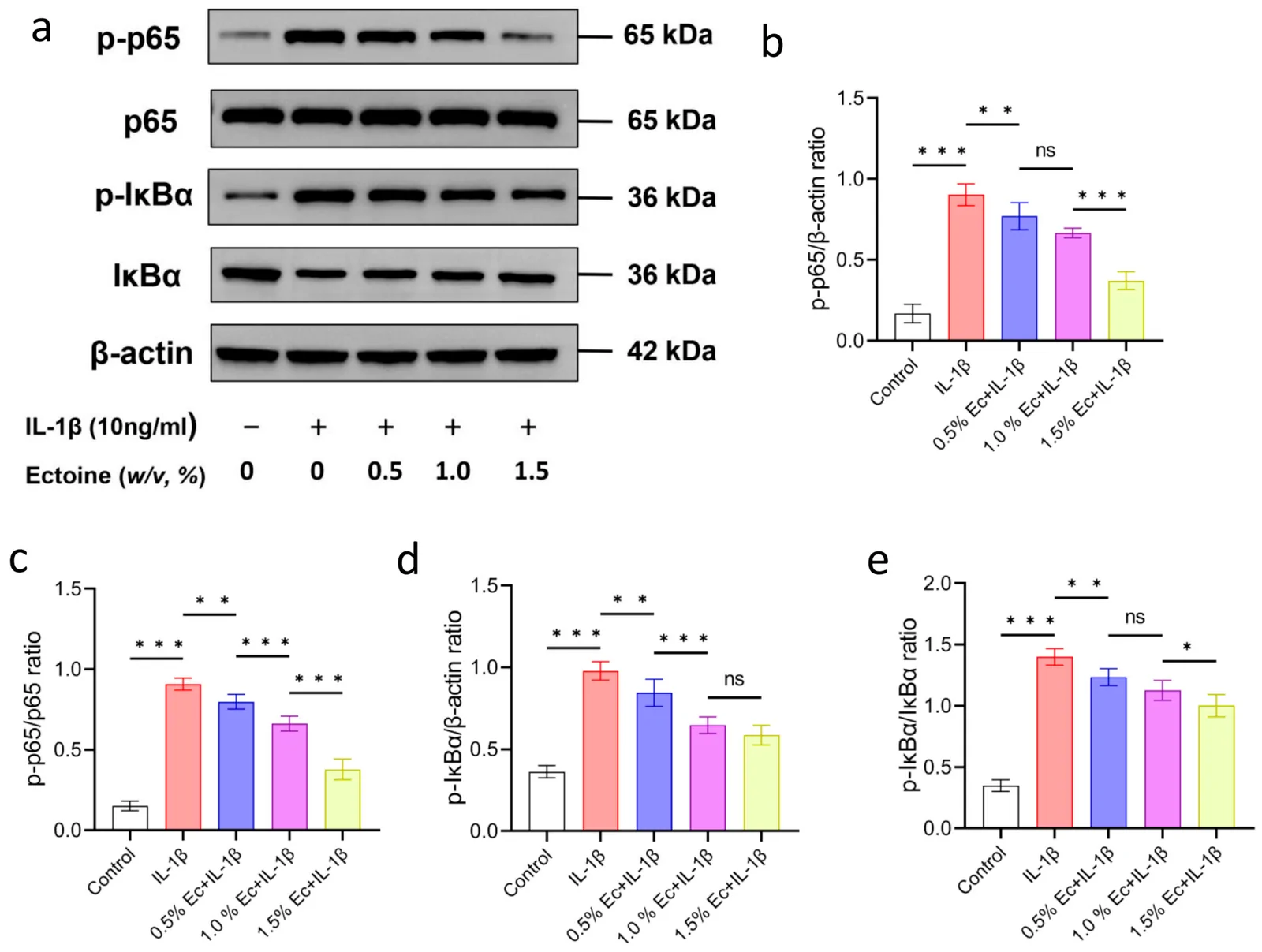
Ectoine suppressed IL-1β-mediated NF-κB pathway activation in chondrocytes. (**a**) Western blots of NF-κB pathway components (p65, p-p65, IκBα, and p-IκBα) following ectoine pretreatment and IL-1β stimulation. Ectoine dose-dependently suppressed IL-1β-induced p65 phosphorylation and IκBα degradation. (**b**–**e**) Quantitative analysis of p65, p-p65, IκBα, and p-IκBα protein levels (the samples derive from the same experiment and that blots were processed in parallel) (*n* ≥ 3, one-way *ANOVA*, ns, *p* > 0.05; * *p* < 0.05, ** *p* < 0.01, *** *p* < 0.001).

**Figure 4:**
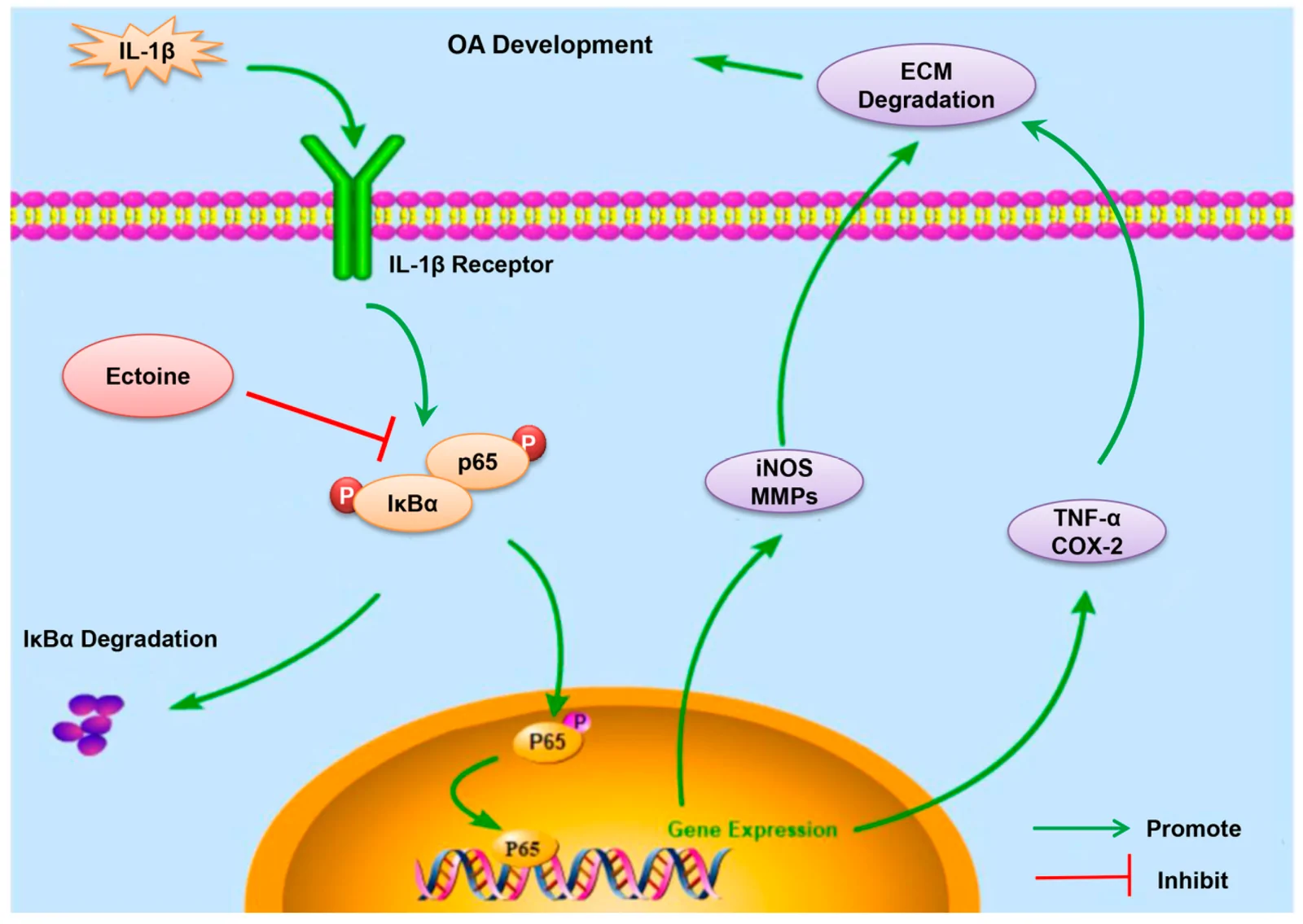
Mechanistic schematic of ectoine-mediated NF-κB suppression and its therapeutic potential in OA pathogenesis. Red arrows indicate inhibitory effects, whereas green arrows represent activation pathways.

The NF-κB pathway, a key regulator of inflammation, was assessed by measuring p65 phosphorylation and IκBα degradation. Compared with the control, IL-1β stimulation markedly increased p65 phosphorylation (*p* < 0.001). Ectoine inhibited this activation in a dose-dependent manner, with 1.5% ectoine reducing p65 phosphorylation by 59% and IκBα degradation by 41% relative to the IL-1β group (*p* < 0.001; Figure 3a–e), indicating its anti-inflammatory mechanism. A schematic of the proposed mechanism is shown in Figure 4.

### 3.4. Ectoine Reduced Cartilage Damage and NF-κB Activation In Vivo

**Figure 5:**
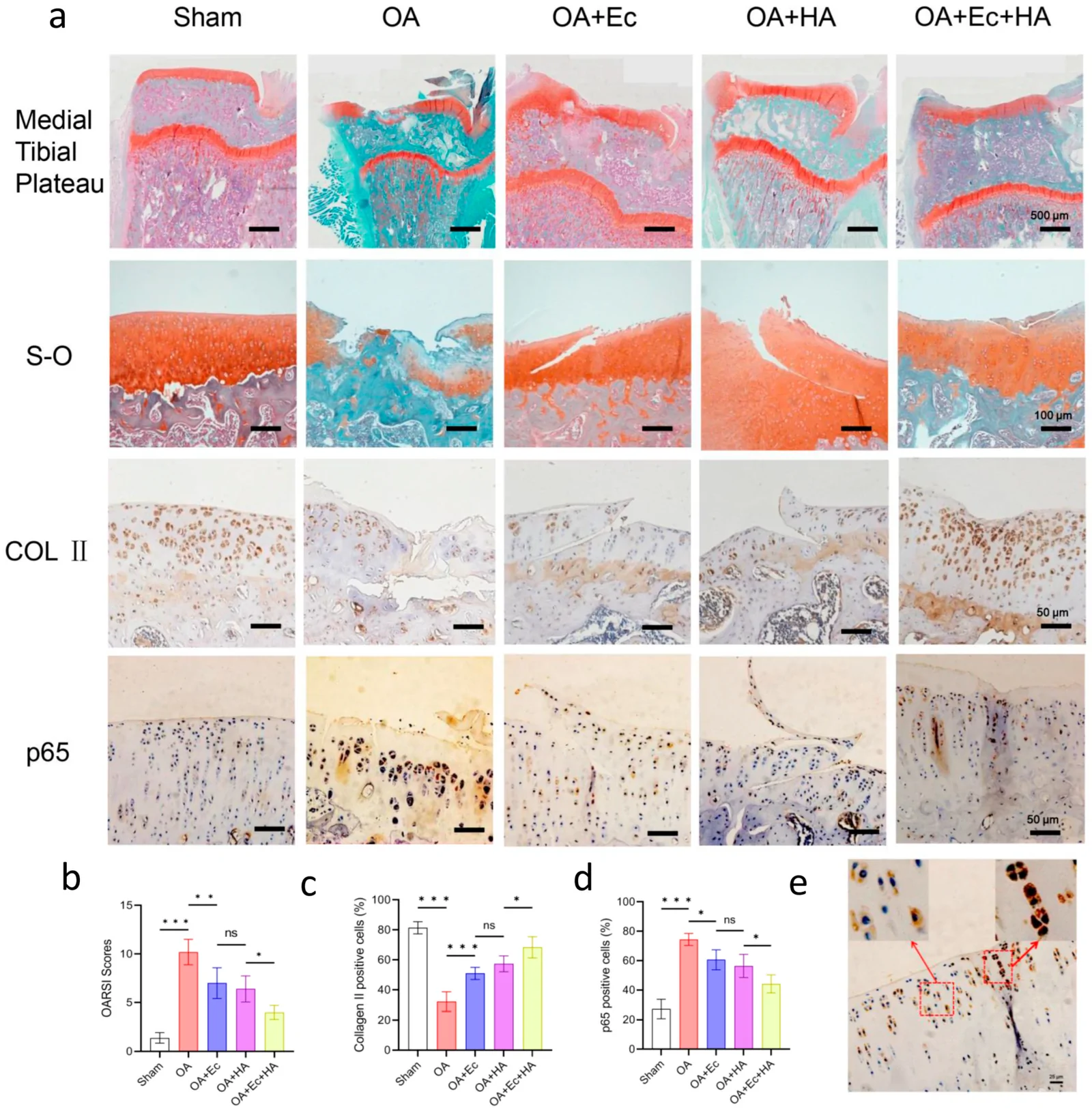
Therapeutic effects of ectoine in a rat OA model. (**a**) Representative sections (S-O) and IHC staining for collagen II and p65 in the medial tibial plateau articular cartilage 8 weeks after surgery. (**b**) OARSI scores of cartilage degeneration. (**c**,**d**) Quantitative analysis of collagen II-positive and p65-positive chondrocytes. (**e**) High-magnification image of p65 IHC staining. Nuclear p65 expression is markedly increased in chondrocytes at cartilage lesion sites. (*n* = 12, one-way *ANOVA*, scale bar: 100/50 μm, ns, *p* > 0.05; * *p* < 0.05, ** *p* < 0.01, *** *p* < 0.001).

In vivo evaluation using a surgically induced rat OA model demonstrated the therapeutic efficacy of ectoine. The OA group exhibited marked cartilage erosion, chondrocyte depletion, and matrix loss, whereas ectoine and HA treatments preserved cartilage integrity, particularly in the Ec + HA group (Figure 5a). OARSI scoring confirmed milder pathological changes in the ectoine group (score: 7.0 ± 1.58) than in the OA group (score: 10.2 ± 1.30). Collagen retention also improved notably, with Ec + HA enhancing it by 43% compared with ectoine alone (Figure 5b).

IHC showed that ectoine and HA preserved type II collagen expression relative to OA (*p* < 0.001), with Ec + HA showing the strongest effects (*p* < 0.05) and fewer cartilage defects (Figure 5c). Moreover, p65 expression, which was superficial in the sham group, increased throughout the cartilage tissue of the OA group. Ectoine and HA monotherapies reduced p65 expression, with the Ec + HA combination showing the most evident reduction (Figure 5d). Close examination confirmed p65 nuclear translocation in damaged cartilages (Figure 5e), consistent with in vitro inhibition of the NF-κB pathway. These findings indicate that Ec slows OA progression by maintaining cartilage structure and suppressing NF-κB, with potential additive benefits when combined with HA.

### 3.5. Ectoine Downregulated Inflammatory Gene Expression in Cartilage

**Figure 6:**
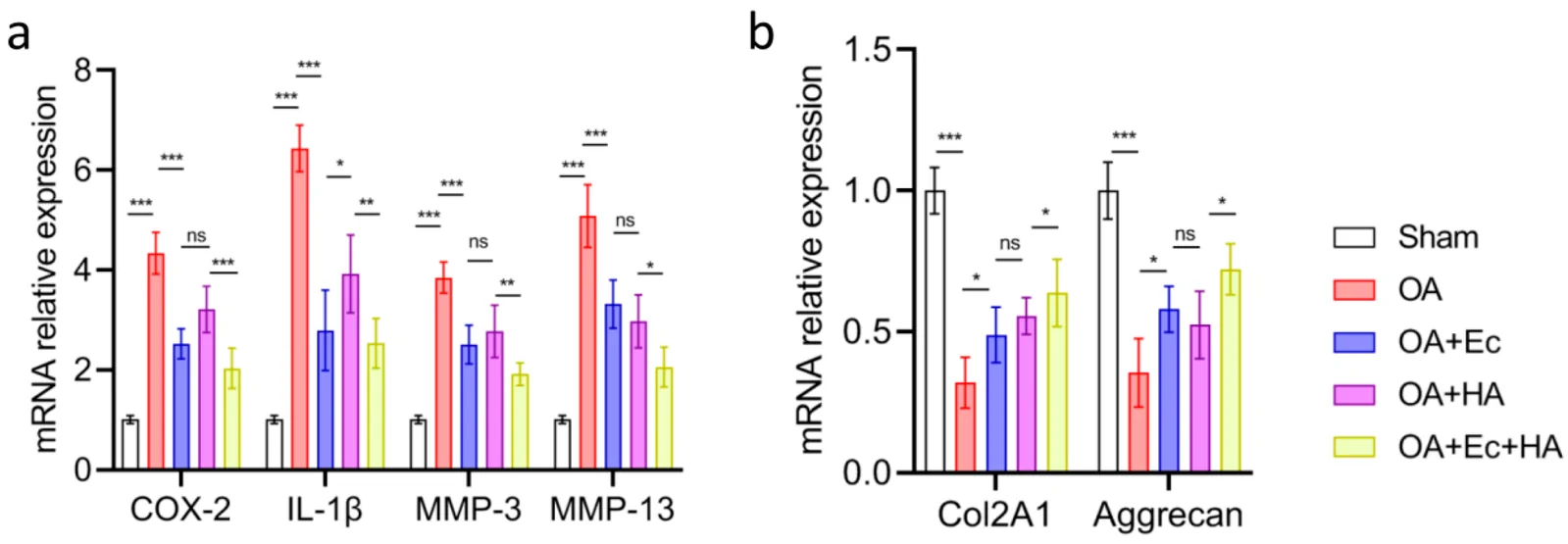
Cartilage tissue gene expression analysis using RT-qPCR. (**a**) Relative mRNA levels of inflammatory mediators (*COX-2*, *IL-1β*, *MMP-3*, and *MMP-13*) in different groups. (**b**) Expression of cartilage matrix components (*Col2A1*, *aggrecan*) in rat articular cartilage. (*n* ≥ 5, one-way *ANOVA*, ns, *p* > 0.05; * *p* < 0.05, ** *p* < 0.01, *** *p* < 0.001).

RT-qPCR of femoral condyle cartilages at 8 weeks post-surgery showed differences in gene expression among groups. Elevated mRNA levels of *COX-2*, *MMP-3*, *MMP-13*, and *IL-1β* were observed in the OA group compared with the treatment groups (*p* < 0.001). Ectoine and HA alone downregulated these markers to a similar extent, whereas the Ec + HA combination showed more pronounced suppression (*p* < 0.05 versus single treatments; Figure 6). Conversely, cartilage matrix genes for *Col2A1* and *aggrecan* showed the opposite pattern. Their expression was lowest in the OA group and highest in the treatment groups (*p* < 0.05), with the Ec + HA combination outperforming monotherapies (*p* < 0.05). These results suggest that ectoine may reduce inflammation and support cartilage matrix maintenance, with greater benefits when combined with HA.

## 4. Discussion

OA progresses through a self-sustaining cycle of inflammation and cartilage breakdown, with NF-κB signaling acting as a central regulator. This study investigated whether ectoine, a natural microbial compound known to reduce epithelial and mucosal inflammation [14,15,16], could interrupt this cycle by modulating IL-1β-induced NF-κB activation. In cellular and rat OA models, ectoine reduced key inflammatory mediators (iNOS, COX-2, and TNF-α), suppressed cartilage-degrading enzymes (MMP-3 and MMP-13), and preserved matrix production. These effects were consistent with its ability to limit nuclear translocation of NF-κB by reducing IκBα phosphorylation. Notably, combining ectoine with HA enhanced these benefits, supporting a dual-target approach that addresses both molecular and mechanical aspects of OA, a concept consistent with current trends toward multimodal therapy.

NF-κB regulates OA-associated inflammation and tissue destruction. IL-1β, a cytokine strongly implicated in OA [17,18], activates NF-κB through IκB kinase (IKK), leading to IκBα phosphorylation and degradation, which enables nuclear translocation of p65/p50 complexes and promotes the upregulation of inflammatory genes and matrix-degrading enzymes [19,20,21,22]. Our findings suggest that ectoine disrupts this pathway at an important step. Its concentration-dependent efficacy (optimal at 1.5%) is consistent with dose-responsive NF-κB inhibition in colitis and respiratory inflammation models [14], suggesting a conserved mechanism across different tissue types. These observations indicate that ectoine may act as both a broad anti-inflammatory agent and an OA-specific NF-κB modulator.

The difference between ectoine and conventional NF-κB inhibitors has not been fully established. Unlike small molecules (e.g., BAY 11-7082) or natural derivatives such as parthenolide and andrographolide, which broadly target IKKs, proteasomes, or upstream signals [23,24,25], our results show that ectoine reduces IκBα degradation and p65 phosphorylation, suggesting that it may interfere with NF-κB activation, potentially by stabilizing the cytoplasmic IκBα–p65 complex. However, direct evidence for this stabilization or molecular specificity is lacking. If confirmed, such a mechanism could help minimize broad immunosuppression while supporting the anabolic functions of chondrocytes. Ectoine also preserved type II collagen and aggrecan synthesis, which are key markers of cartilage repair [26,27], potentially reactivating native repair mechanisms following NF-κB inhibition. For instance, 1.5% ectoine-induced Col2A1 upregulation is consistent with reports that NF-κB inhibition restores SRY-box transcription factor 9 activity, a key transcription factor for collagen synthesis [28]. Thus, ectoine may contribute to both reducing breakdown and supporting cartilage matrix renewal.

In rat studies, Ec + HA reduced cartilage damage more effectively than ectoine alone. The mechanisms underlying this enhanced effect remain unclear, but they may involve improved joint lubrication, prolonged retention of ectoine in the joint space, or complementary actions on cartilage metabolism [29]. This finding is consistent with current clinical approaches that favor multimodal OA therapies [30]. The observed reduction in cartilage damage supports the translational potential of this combination, particularly for early-stage OA, in which biomechanical support may slow progression.

In this study, ectoine treatment was started at 3 weeks after surgery rather than immediately after OA induction. This timing was selected to assess the therapeutic effects of ectoine in early-to-mild-stage OA rather than its preventive potential. Previous studies using the modified Hulth method have shown that early osteoarthritic changes appear as early as 4–6 weeks after surgery, with significant pathological changes evident by 8 weeks [31,32]. By 3 weeks after surgery, the OA process had already been initiated, and mild pathological changes were observed in this study. Thus, treatment initiation at this time simulated a clinical scenario in which patients with mild symptomatic OA receive intervention. We acknowledge that earlier intervention may produce different outcomes; future studies comparing treatment windows, including preventive, early-stage, and late-stage interventions, would help clarify the optimal timing for ectoine in OA management.

The potential advantages of ectoine over existing NF-κB-targeting treatments should be considered. Conventional anti-inflammatories, such as glucocorticoids, reduce inflammation but may worsen cartilage damage over time [33]. Biologics, such as anti-TNF-α antibodies, target single downstream factors without promoting repair and have risks related to high cost and immunogenicity [34]. In contrast, ectoine, a naturally derived compound, has high solubility, stability, and biocompatibility, making it suitable for local joint application with limited systemic effects. As a protective molecule in microbes, ectoine is known to stabilize proteins and membranes under stress [6]. Although these broad cytoprotective properties may contribute to its benefits, our data are consistent with a mechanism involving reduced IκBα degradation and p65 phosphorylation, which helps regulate NF-κB activation. Nonetheless, the anti-osteoarthritic effects of ectoine are likely multifactorial, and future research should examine potential roles of other stress-responsive pathways (e.g., mitogen-activated protein kinase [MAPK] and NF erythroid 2-related factor 2 [Nrf2]).

The safety profile of ectoine also supports its consideration as a candidate for long-term OA management. Unlike proteasome inhibitors, which disrupt normal protein turnover, if ectoine indeed stabilizes the IκBα–p65 complex, it may preserve baseline NF-κB activity essential for cellular function. This selectivity likely explains why ectoine-treated chondrocytes remained viable and functionally active, providing an important advantage for chronic conditions such as OA.

Nevertheless, OA is a heterogeneous disorder that extends beyond IL-1β/NF-κB signaling and cartilage degradation. Clinical outcomes are influenced by pain processing, metabolic factors, and individual patient characteristics, and structural preservation in animal models does not necessarily predict pain relief or functional improvement in humans [35]. Therefore, our preclinical findings should not be overinterpreted as evidence of clinical efficacy; future studies must evaluate ectoine within a multimodal framework using patient-relevant endpoints.

Because these findings are preclinical and model-specific, they may not directly translate to human OA. Intra-articular ectoine is not an established OA therapy; its optimal dose, pharmacokinetics, safety after repeated administration, and long-term effects remain unknown. Nonetheless, the results suggest two main implications for future research. First, the established safety of ectoine in humans for other inflammatory conditions supports its repurposing for joint injection, thereby avoiding the systemic side effects of oral NSAIDs. Second, its synergy with HA may support the development of combination products, such as hydrogels or sustained-release formulations, to prolong ectoine retention in the joint. For instance, incorporating ectoine into thermosensitive hydrogels could mimic the natural synovial fluid retention of HA [36], although experimental validation is required.

We acknowledge several limitations in this study. First, the relatively short observation period prevents assessment of the sustained long-term effects of ectoine, and the sample size of 12 rats per group, although sufficient to detect major differences, may not have enough power to identify modest treatment effects; confirmation in larger cohorts is therefore warranted. Second, although the surgically induced OA model recapitulates key structural features of human OA, it does not fully reproduce the complex pathophysiology of the human disease. In addition, our in vitro experiments were performed on chondrocytes derived from juvenile rats, which may have metabolic and inflammatory responses different from those of adult or human chondrocytes; thus, validation in more physiologically relevant models, including human cells or aged animals, is required before clinical translation. Third, while our data suggest that ectoine inhibits NF-κB activation by reducing IκBα phosphorylation and p65 nuclear translocation, the precise molecular targets remain unknown; possible upstream mechanisms, such as direct IKK modulation or involvement of other regulators, require further investigation. Other stress-responsive pathways, including MAPK and Nrf2, may also contribute to the observed effects. Fourth, the addition of 1.5% (*w*/*v*) ectoine (~106 mM) increased the medium osmolarity from approximately 336 mOsm/kg (DMEM with 10% FBS) to approximately 436 mOsm/kg, which may induce non-specific osmotic responses. Moreover, no osmotic control (e.g., NaCl) was included in this study. Therefore, the anti-inflammatory effects may be partly attributable to hyperosmolarity rather than to the specific biological activity of ectoine. Finally, our evidence for p65 nuclear translocation is indirect, being mainly based on IHC staining and Western blotting of whole-cell lysates. Consequently, the conclusion that ectoine suppresses NF-κB signalling by blocking p65 nuclear translocation should be interpreted cautiously and requires confirmation using more direct experimental approaches in future studies. Given these limitations, future investigations should focus on clarifying the precise mechanisms, optimising delivery strategies, evaluating long-term safety, and exploring synergistic combinations with other therapeutic agents.

## 5. Conclusions

In conclusion, in this preclinical rat model, ectoine was associated with reduced markers of NF-κB activation, attenuated inflammatory and catabolic responses in chondrocytes, and ameliorated OA-like damage in a rat model. Its enhanced effect with HA suggests that combination therapies may warrant further investigation. Although these findings highlight the potential of ectoine in OA management, further research is required to clarify its mechanisms and translational applications.

## Figures and Tables

**Table 1:** Primer sequences for RT-qPCR.

Gene	Primer Sequence
*iNOS*	F: 5′-GCTGAACTTGAGCGAGGA-3′
	R:5′-ACTCAGTGCCAGAAGCTGGA-3′
*COX-2*	F: 5′-AAAGGCCTCCATTGACCAGA-3′
	R:5′-TCGATGTCATGGTAGAGGGC-3′
*TNF-α*	F: 5′-GGCGGTGCTCGCTTTGT-3′
	R:5′-GCCCTGTATTCCGTCTCCTT-3′
*MMP-3*	F: 5′-TTTGGCCGTCTCTTCCATCC-3′
	R: 5′-GCATCGATCTTCTGGACGGT-3′
*MMP-13*	F: 5′-TCCTGATGATGATGTGCAAGG-3′
	R: 5′-TTGTCTGGTGTTTTGGGGTGT-3′
*IL-1β*	F: 5′-GCACAGTTCCCCAACTGGTA-3′
	R: 5′-GGAGACTGCCCATTCTCGAC-3′
*Aggrecan*	F: 5′-CTCCAATGACTCCGGGATCTAC-3′
	R: 5′-CCTTTCACCACCACCTCCA-3′
*Col2A1*	F: 5′-GGCAATAGCAGGTTCACGTACA-3′
	R: 5′-GATAACAGTCTTGCCCCACTTACC-3′
*β-actin*	F: 5′-GGGAAATCGTGCGTGAC-3′
	R: 5′-TTGCCAATGGTGATGACCTG-3′

## Data Availability

The datasets supporting the conclusions of this article are available in the Harvard Dataverse repository, https://doi.org/10.7910/DVN/XU0WVI.

## Abbreviations

The following abbreviations are used in this manuscript:

Col2A1	Collagen type II alpha-1
Ec	Ectoine
HA	Hyaluronic acid
IHC	Immunohistochemistry
IκBα	Inhibitor of nuclear factor kappa-B alpha
IKK	IκB kinase
IL-1β	Interleukin-1β
iNOS	Inducible nitric oxide synthase
MAPK	Mitogen-activated protein kinase
MTT	3-(4,5-dimethylthiazol-2-yl)-2,5-diphenyltetrazolium bromide
NF-κB	Nuclear factor-κB
Nrf2	Nuclear factor erythroid 2-related factor 2
TNF-α	Tumor necrosis factor-α
